# Supplementary material for: GloPath: An Entity‐Centric Foundation Model for Glomerular Lesion Assessment and Clinicopathological Insights
Source: Adv Sci (Weinh). 2026 Apr 15;13(29):e20580. doi: 10.1002/advs.202520580 (PMC13205756; doi:10.1002/advs.202520580)
Supplement: Supplementary file 1 — Supporting File: advs74818‐sup‐0001‐SuppMat.pdf. [file ADVS-13-e20580-s001.pdf]

Supplementary materials

Menu of supplementary content

1. Supplementary Figures

Fig. S1 Comparison of IoU of all the models on morphological segmentation, which shows the results of bow segmentation on XJ-Light and tuft segmentation on XJ-GIO.
Fig. S2 Comparison of segmentation visualization of all the models. a, Performance of bow segmentation. Column 1-9 indicate original images, ground truth, results of CONCH, GloPath, RenalPath, ImageNetPre, RandomInit, PLIP and UNI. The masks represent the target areas. b, Performance of tuft segmentation.
Fig. S3 Performance of all the compared models on few-shot leanring on AIDPATH-G with confidence intervals.
Fig. S4 Performance of all the compared models on few-shot leanring on XJ-IF with confidence intervals.
Fig. S5 Clinicopathological correlation analysis on XJ-Light-1. The x-axis represents clinical parameters and their respective groups, while the y-axis shows the statistical distribution of pathological phenotypes within each group.
Fig. S6 Clinicopathological correlation analysis on KPMP-G. The definitions of the x-axis and y-axis are the same as in Fig. S5.
Fig. S7 Examples of the XJ-Light dataset. a, Examples of slides from 12 levels. b, Examples of glomeruli from four stainings.
Fig. S8 Interface of the Kidney Label Tool which is used for lesion annotation.
Fig. S9 Examples of the XJ-IF dataset with different deposition regions and patterns and with diverse markers.
Fig. S10 Examples of the AIDPATH-G dataset. a, Renal pathology images and their annotations. b, Examples of normal and sclerotic glomeruli.
Fig. S11 Examples of slides and extracted glomeruli with different stainings in the KPMP-G dataset.
Fig. S12 Error analysis of GloPath on XJ-CLI.
Fig. S13 UMAP projection across different weights and imaging domains.
Fig. S14 Interpretability study of GloPath.

2. Supplementary Tables

Table S1 Details of the 52 lesion assessment tasks.
Table S2 Number of glomeruli with each type of lesion in XJ-CLI.
Table S3 Comparison of methods on lesion recognition in PAS staining (F1 score). The bold value indicates the best model.
Table S4 Comparison of methods on lesion recognition in MT staining (F1 score). The bold value indicates the best model.
Table S5 Comparison of methods on lesion recognition in PASM staining (F1 score). The bold value indicates the best model.
Table S6 Comparison of methods on AIDPATH-G under full supervision. The bold value indicates the best model.
Table S7 Comparison of methods on AIDPATH-G using SVM-based few-shot learning. The bold value indicates the best model.
Table S8 Comparison of methods on AIDPATH-G using LR-based few-shot learning. The bold value indicates the best model.
Table S9 Comparison of methods on AIDPATH-G using MLP-based few-shot learning. The bold value indicates the best model.
Table S10 Comparison of methods on AIDPATH-G using RF-based few-shot learning. The bold value indicates the best model.
Table S11 Comparison of methods on AIDPATH-G using PTL-based few-shot learning. The bold value indicates the best model.
Table S12 Comparison of methods on lesion grading (ROC-AUC). The bold value indicates the best model.

Table S13 Comparison of methods on semantic segmentation of the glomerular bow and tuft. The bold value indicates the best model.
Table S14 Comparison of significance level of pathomics-data mining of XJ-Light-1 for GloPath, UNI, and RenalPath.
Table S15 Comparison of significance level of pathomics-data mining of KPMP-G for GloPath, UNI, and RenalPath.
Table S16 Significance of clinicopathological correlation analysis for Gender on XJ-Light-1 (p-value).
Table S17 Significance of clinicopathological correlation analysis for Age on XJ-Light-1 (p-value).
Table S18 Significance of clinicopathological correlation analysis for Creatinine on XJ-Light-1 (p-value).
Table S19 Significance of clinicopathological correlation analysis for IgA (Binary) on XJ-Light-1 (p-value).
Table S20 Significance of clinicopathological correlation analysis for IgA Lee Score on XJ-Light-1 (p-value).
Table S21 Significance of clinicopathological correlation analysis for Disease on XJ-Light-1 (p-value).
Table S22 Significance of clinicopathological correlation analysis for Lesion on XJ-Light-1 (p-value).
Table S23 Multivariate Regression Analysis of Pathology Phenotypes Associated with Creatinine.
Table S24 Multivariate Regression Analysis of Pathology Phenotypes Associated with Disease Type.
Table S25 Significance of clinicopathological correlation analysis for Enrollment Category on KPMP-G (p-value).
Table S26 Significance of clinicopathological correlation analysis for Gender on KPMP-G (p-value).
Table S27 Significance of clinicopathological correlation analysis for Age on KPMP-G (p-value).
Table S28 Significance of clinicopathological correlation analysis for Proteinuria on KPMP-G (p-value).
Table S29 Significance of clinicopathological correlation analysis for A1c on KPMP-G (p-value).
Table S30 Significance of clinicopathological correlation analysis for Albuminuria on KPMP-G (p-value).
Table S31 Significance of clinicopathological correlation analysis for Diabetes History on KPMP-G (p-value).
Table S32 Significance of clinicopathological correlation analysis for Hypertension History on KPMP-G (p-value).
Table S33 Significance of clinicopathological correlation analysis for eGFR on KPMP-G (p-value).
Table S34 Comparison of methods on XJ-IF for cross-modality diagnosis based on full supervision.
Table S35 Comparison of methods on deposition region classification on XJ-IF using LR-based few-shot learning. The bold value indicates the best model and the hyphen mean the metrics value is lower than 0.5 and not applicable.
Table S36 Comparison of methods on deposition pattern classification on XJ-IF using LR-based few-shot learning. The bold value indicates the best model and the hyphen mean the metrics value is lower than 0.5 and not applicable.
Table S37 Comparison of methods on deposition region classification on XJ-IF using MLP-based few-shot learning. The bold value indicates the best model and the hyphen mean the metrics value is lower than 0.5 and not applicable.
Table S38 Comparison of methods on deposition pattern classification on XJ-IF using MLP-based few-shot learning. The bold value indicates the best model and the hyphen mean the metrics value is lower than 0.5 and not applicable.
Table S39 Comparison of methods on deposition region classification on XJ-IF using RF-based few-shot learning. The bold value indicates the best model and the hyphen mean the metrics value is lower than 0.5 and not applicable.
Table S40 Comparison of methods on deposition pattern classification on XJ-IF using RF-based few-shot learning. The bold value indicates the best model and the hyphen mean the metrics value is lower than 0.5 and not applicable.
Table S41 Comparison of methods on deposition region classification on XJ-IF using PTL-based few-shot learning. The bold value indicates the best model and the hyphen mean the metrics value is lower than 0.5 and not applicable.
Table S42 Comparison of methods on deposition pattern classification on XJ-IF using PTL-based few-shot learning. The bold value indicates the best model and the hyphen mean the metrics value is lower than 0.5 and not applicable.
Table S43 Performance of GloPath in large-scale real-world study.
Table S44 Details of the glomerular lesion annotation on XJ-Light-1.
Table S45 Details of the IF markers in XJ-IF.

Table S46 Details of the annotated images on XJ-IF.
Table S47 Details of the clinical variables on XJ-Light-1.
Table S48 Details of the clinical variables on KPMP-G.
Table S49 Details of the morphological variables.

1. Supplementary Figures

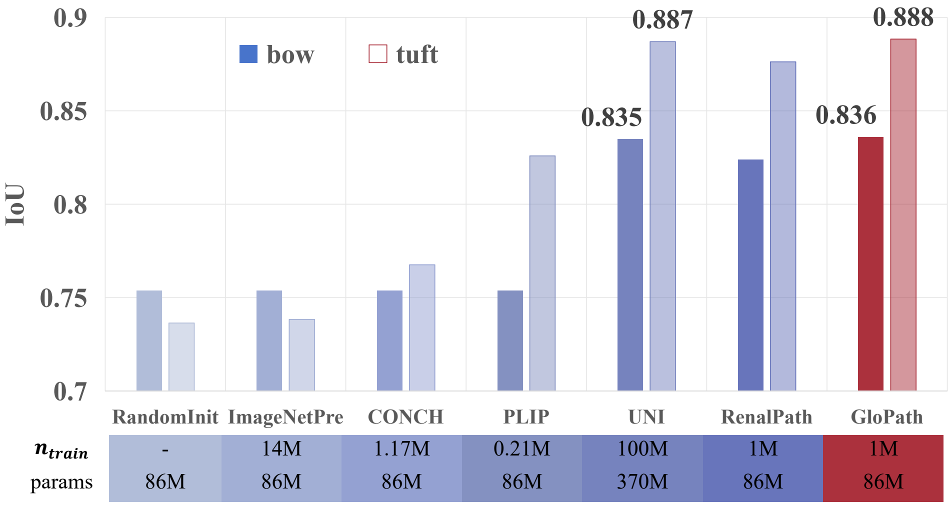

Figure S1: Comparison of IoU of all the models on morphological segmentation, which shows the results of bow segmentaion on XJ-Light and tuft segmentation on XJ-GIO.

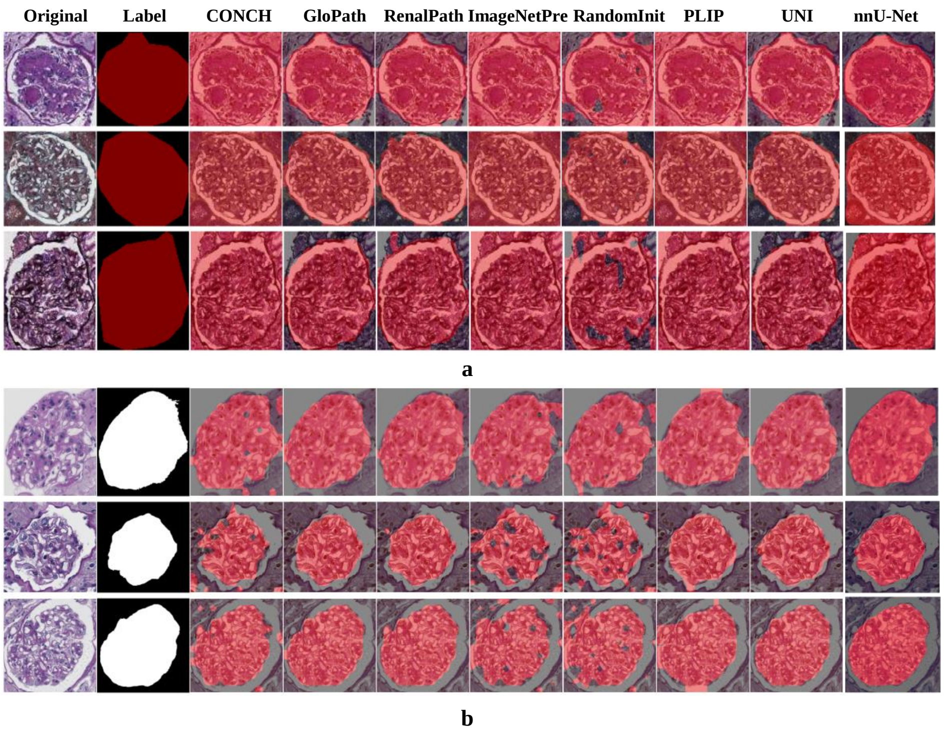

Figure S2: Comparison of segmentation visulization of all the models. **a,** Performance of bow segmentaion. Column 1-10 indicate original iamegs, ground truth, results of CONCH, GloPath, RenalPath, ImageNetPre, RandomInit, PLIP, UNI and nnU-Net. The masks represent the target areas. **b,** Performance of tuft segmentaion.

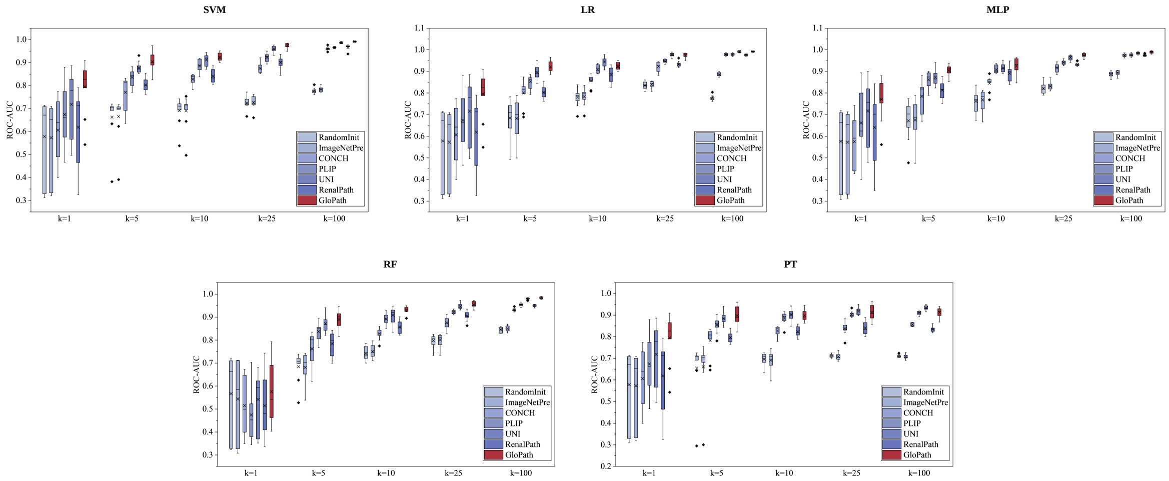

Figure S3: Performance of all the compared models on few-shot leanring on AIDPATH-G with confidence intervals.

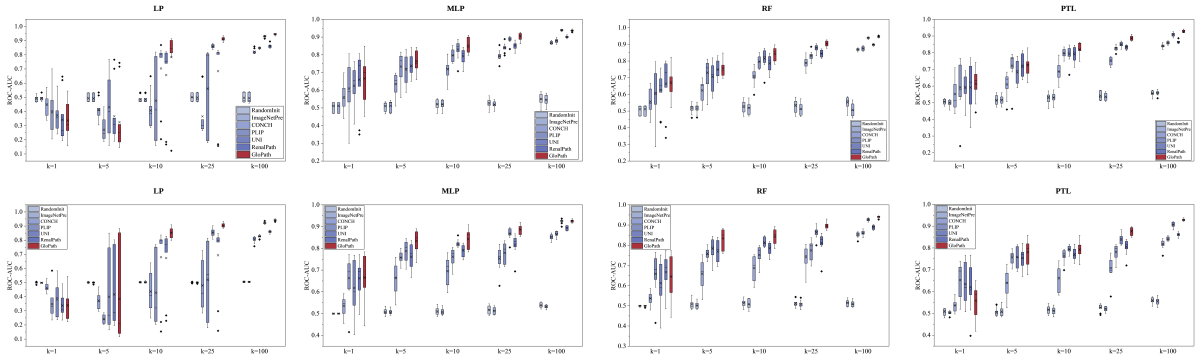

Figure S4: Performance of all the compared models on few-shot leanring on XJ-IF with confidence intervals. Row 1-2 indicate region and pattern classification tasks respectively.

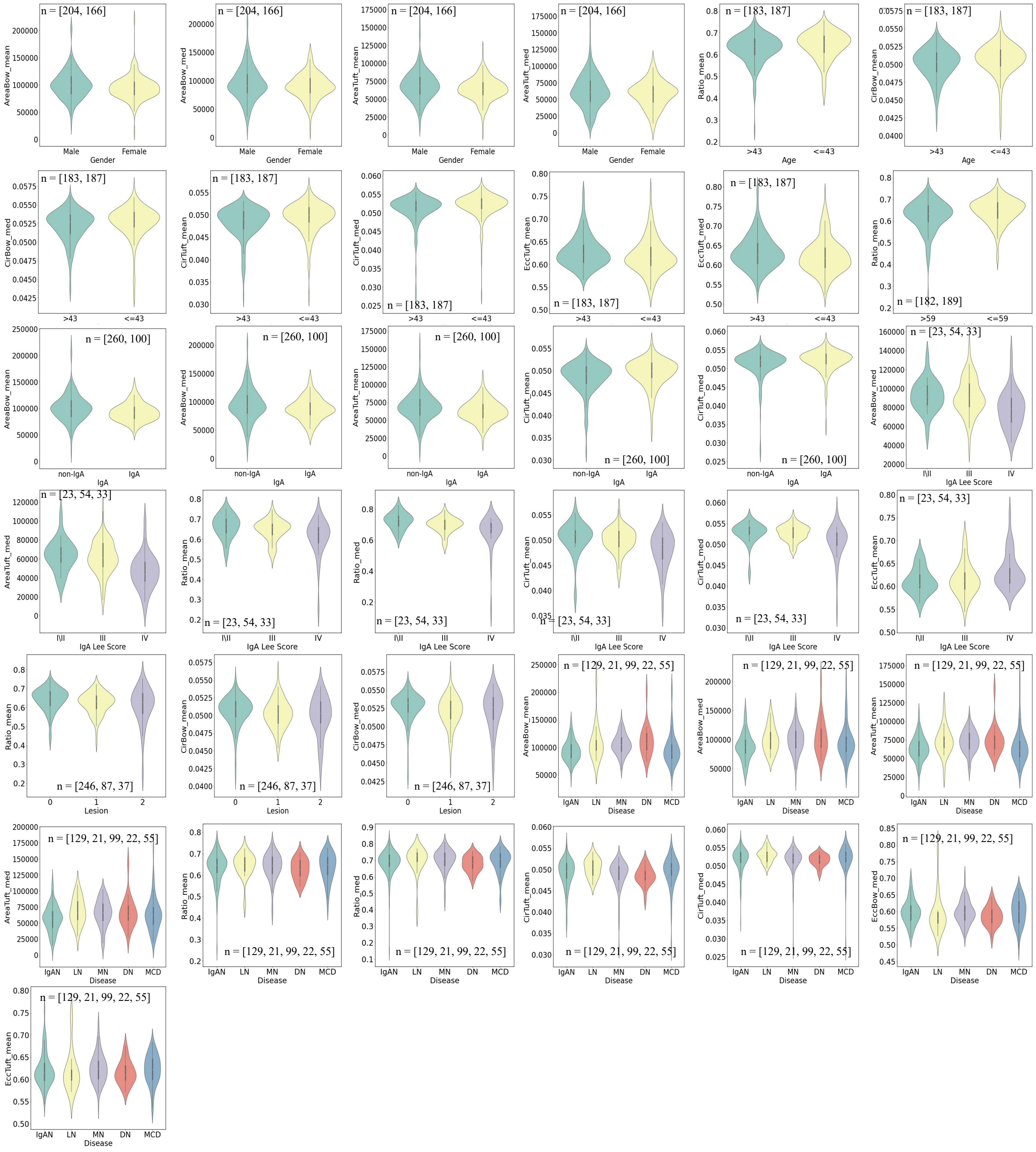

Figure S5: Clinicopathological correlation analysis on XJ-Light-1. The x-axis represents clinical parameters and their respective groups, while the y-axis shows the statistical distribution of pathological phenotypes within each group. Exact P values are provided in Table S16-S22.

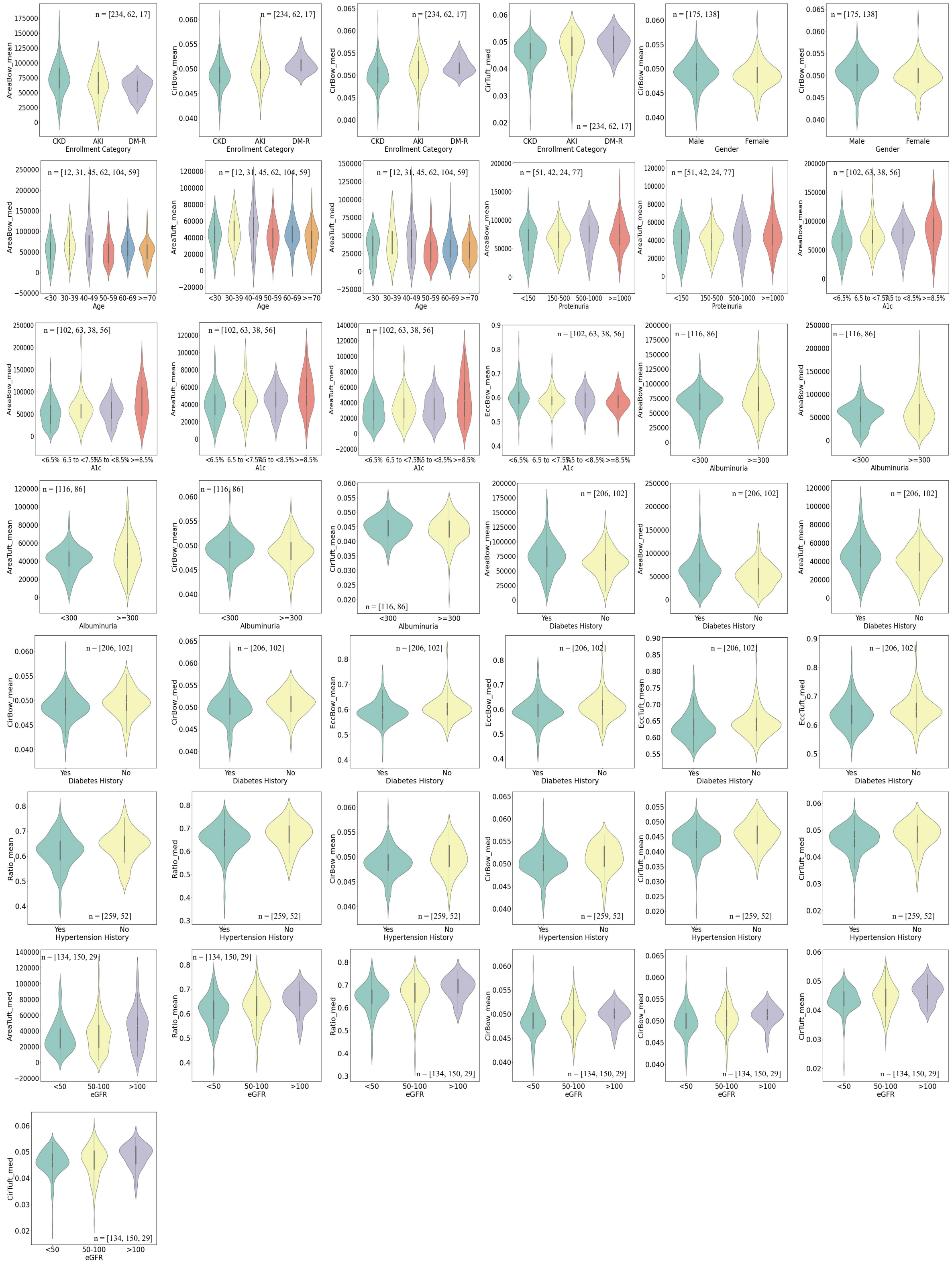

Figure S6: Clinicopathological correlation analysis on KPMP-G. The definitions of the x-axis and y-axis are the same as in Fig. S5. Exact P values are provided in Table S25-S33.

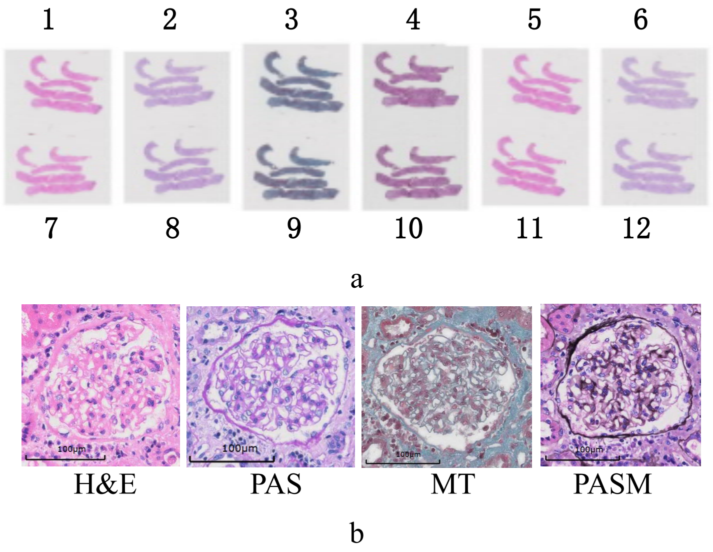

Figure S7: Examples of the XJ-Light dataset. **a,** Examples of slides from 12 levels. **b,** Examples of glomeruli from four stainings.

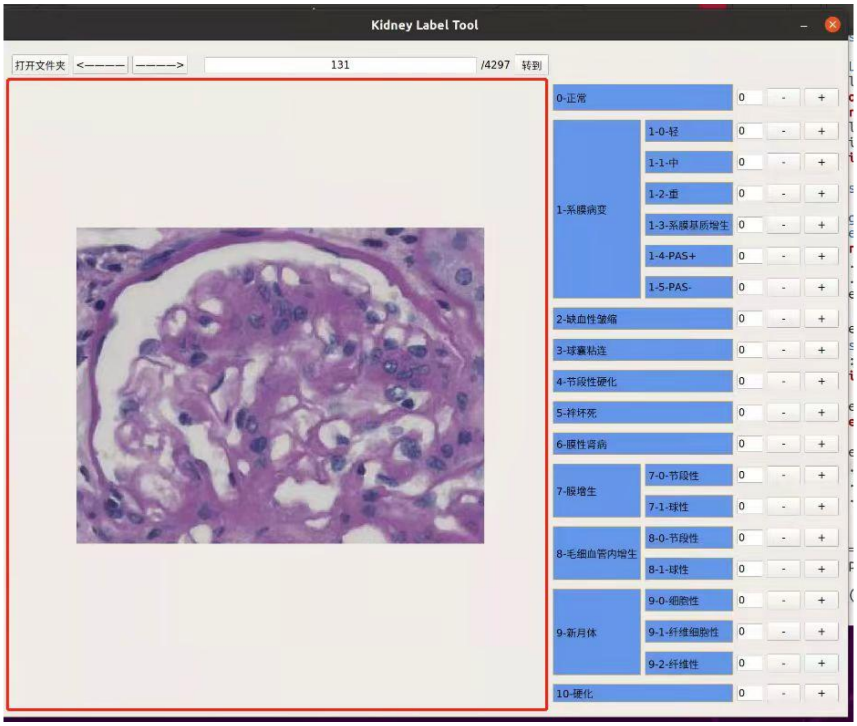

Figure S8: Interface of the Kidney Label Tool which is used for lesion annotation.

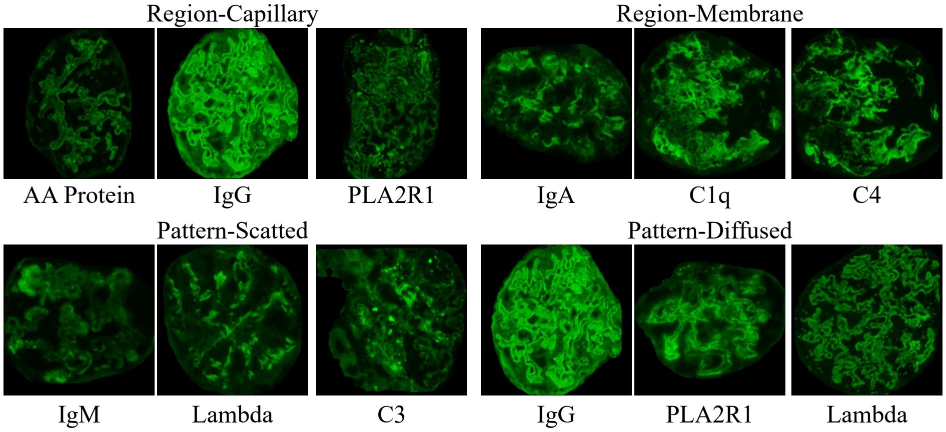

Figure S9: Examples of the XJ-IF dataset with different deposition regions and patterns and with diverse markers.

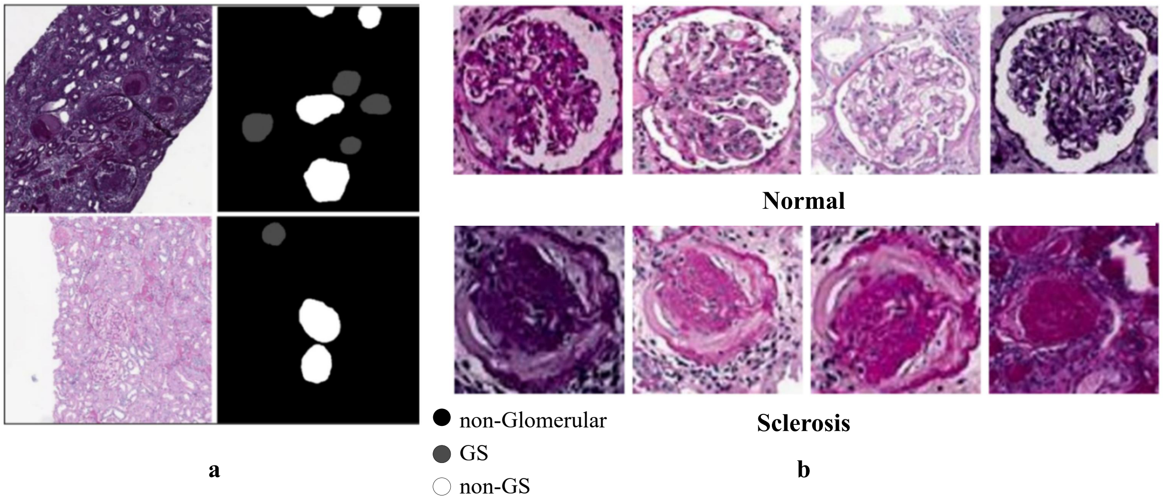

Figure S10: Examples of the AIDPATH-G dataset. **a,** Renal pathology images and their annotations. **b,** Examples of normal and sclerotic glomeruli.

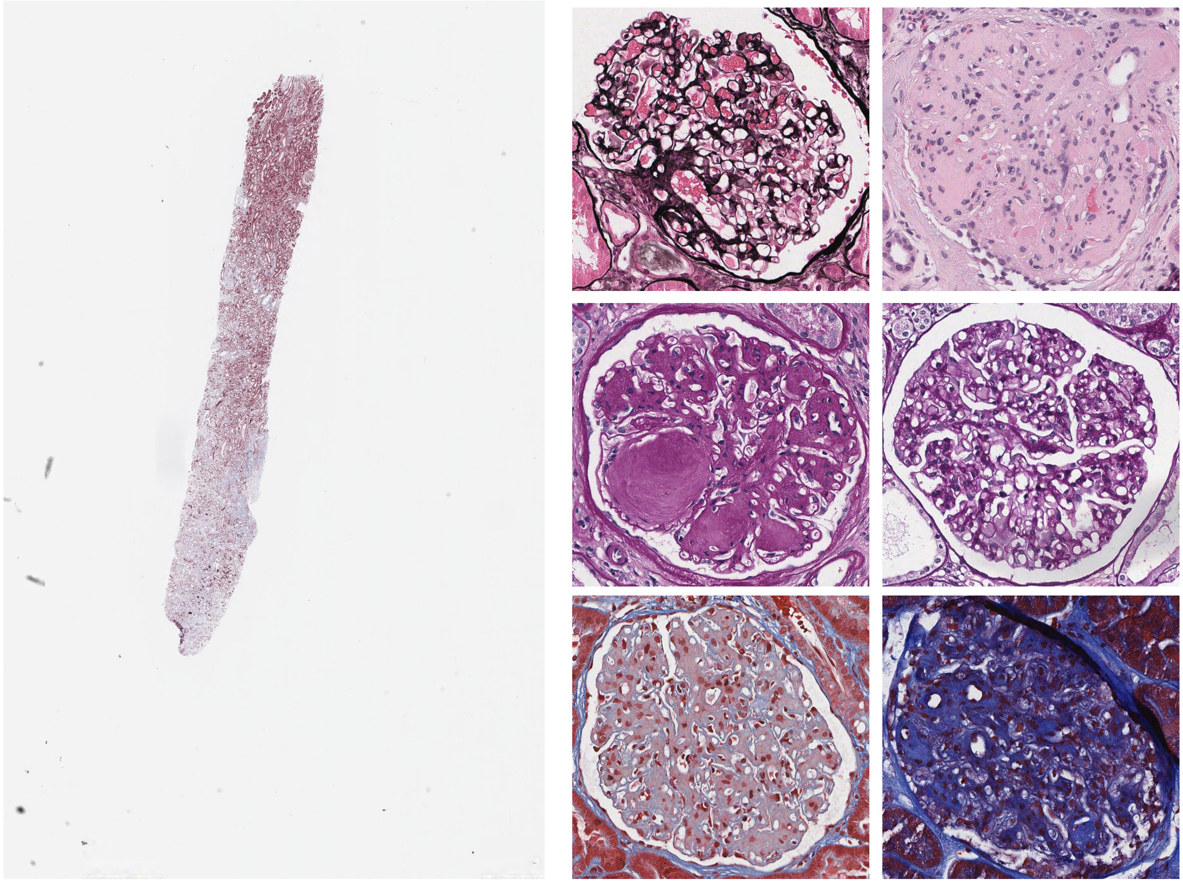

Figure S11: Examples of slides and extracted glomeruli with different stainings in the KPMP-G dataset.

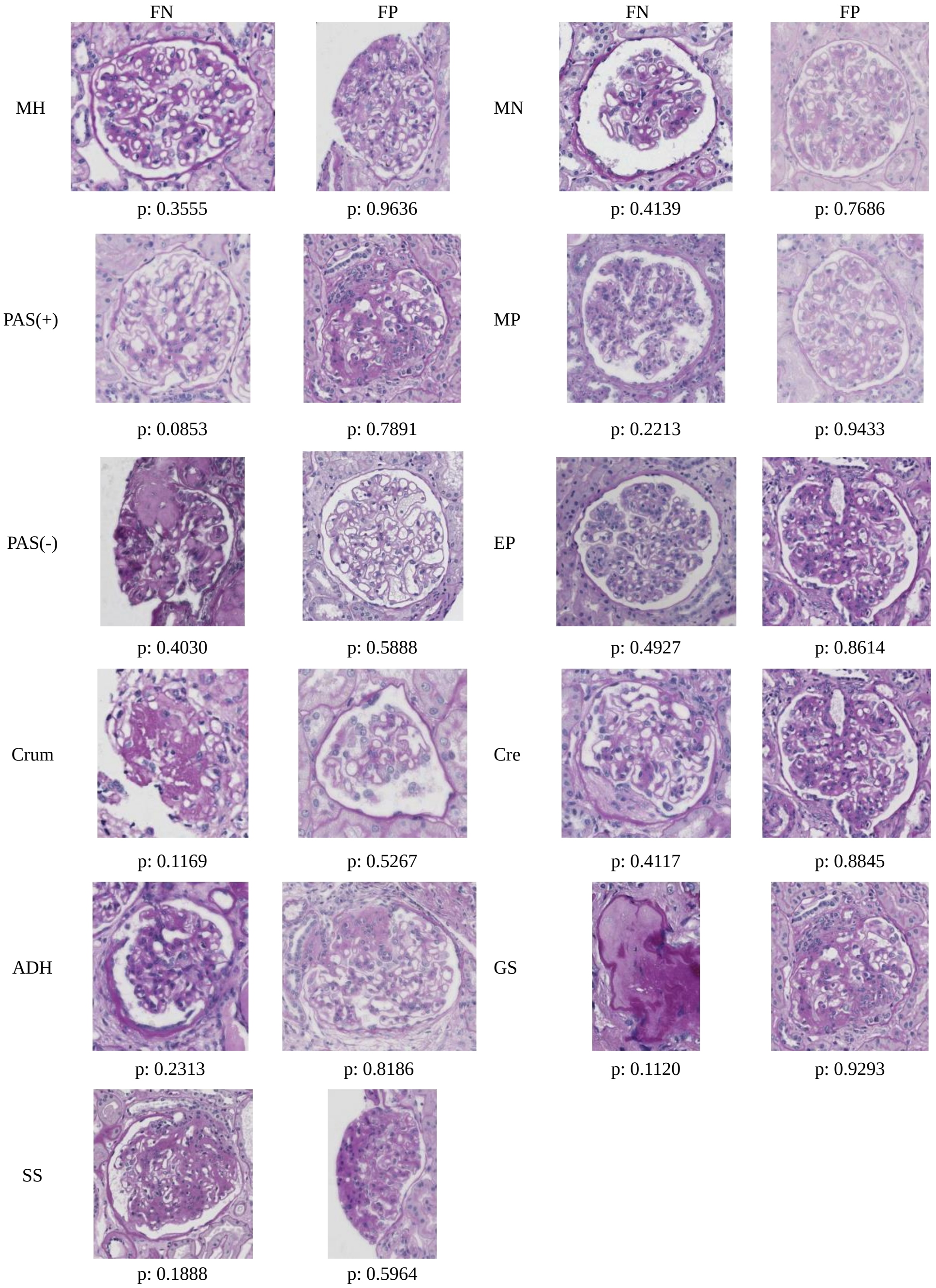

Figure S12: Error analysis of GloPath on XJ-CLI. For each lesion type, we present one false negative and one false positive example, with GloPath's predicted probability for the lesion displayed below each glomerular image (a probability $p > 0.5$ indicates the presence of the lesion).

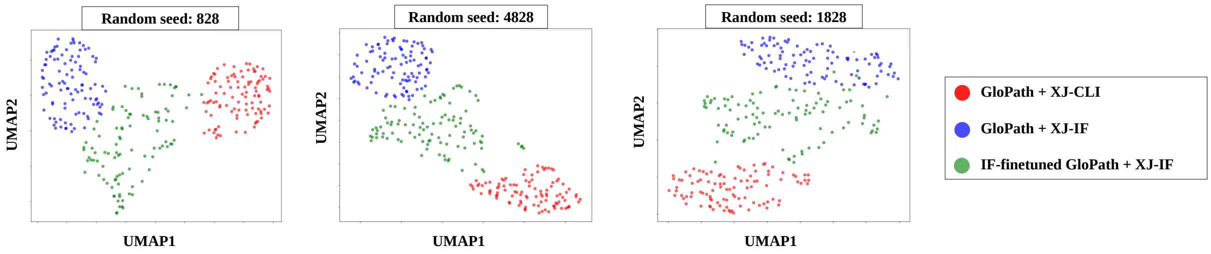

Figure S13: UMAP projection across different weights and imaging domains.

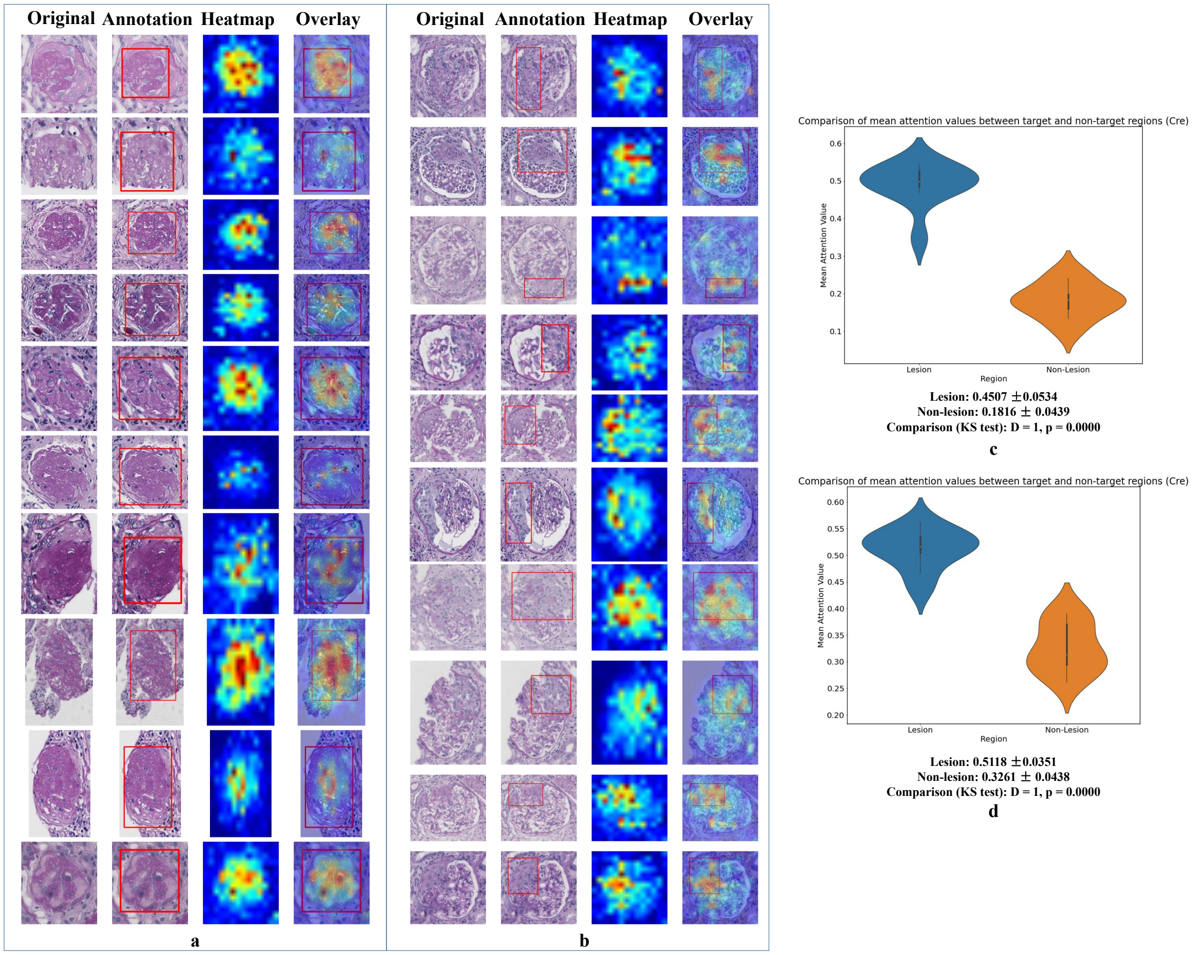

Figure S14: Interpretability study of GloPath. **a,** Comparison between heatmaps generated from GloPath predictions and annotations by nephrologists (GS). Columns 1-4 indicate original images, original images with annotations, heatmaps, and overlays. **b,** Comparison between heatmaps generated from GloPath predictions and annotations by nephrologists (Cre). **c-d,** Comparison of mean attention values between target (lesion) and non-target (non-lesion) regions for GS and Cre, respectively. KS-test results (KS D and p-value) are shown below the violin plots.

2. Supplementary Tables

Table S1: Details of the 52 lesion assessment tasks.

Lesion Recognition	
PAS-MH recognition	Recognition of thylakoid hyperplasia in PAS staining
PAS-PAS(+) recognition	Recognition PAS positivity in PAS staining
PAS-PAS(-) recognition	Recognition PAS negativity in PAS staining
PAS-Crum recognition	Recognition of the presence of ischemic crumpling in PAS staining
PAS-ADH recognition	Recognition the presence of adhesions in PAS staining
PAS-SS recognition	Recognition the presence of segmental sclerosis in PAS staining
PAS-MN recognition	Recognition the presence of glomerular lesions in membranous nephropathy in PAS staining
PAS-MP recognition	Recognition of the presence of glomerulopathy in membranoproliferative glomerulonephritis in PAS staining
PAS-EP recognition	Recognition of the presence of endocapillary proliferation in PAS staining
PAS-Cre recognition	Recognition of the presence of crescents in PAS staining
PAS-GS recognition	Recognition of the presence of global sclerosis in PAS staining
MT-MH recognition	Recognition of thylakoid hyperplasia in MT staining
MT-ADH recognition	Recognition the presence of adhesions in MT staining
MT-SS recognition	Recognition the presence of segmental sclerosis in MT staining
MT-SFN recognition	Recognition the presence of glomerular lesions in membranous nephropathy in MT staining
MT-Cre recognition	Recognition of the presence of crescents in MT staining
MT-GS recognition	Recognition of the presence of global sclerosis in MT staining
PASM-MH recognition	Recognition of thylakoid hyperplasia in PASM staining
PASM-PAS(-) recognition	Recognition PAS negativity in PASM staining
PASM-ADH recognition	Recognition the presence of adhesions in PASM staining
PASM-SS recognition	Recognition the presence of segmental sclerosis in PASM staining
PASM-SFN recognition	Recognition the presence of segmental fibrinoid necrosis in PAS staining
PASM-MN recognition	Recognition the presence of glomerular lesions in membranous nephropathy in PASM staining
PASM-MP recognition	Recognition of the presence of glomerulopathy in membranoproliferative glomerulonephritis in PASM staining
PASM-EP recognition	Recognition of the presence of endocapillary proliferation in PASM staining
PASM-Cre recognition	Recognition of the presence of crescents in PASM staining
PASM-GS recognition	Recognition of the presence of global sclerosis in PASM staining
Lesion Grading	
PAS-MH grading	Classification of PAS-MH as mild, moderate and severe
MT-MH grading	Classification of MT-MH as mild, moderate and severe
PASM-MH grading	Classification of PASM-MH as mild, moderate and severe
PAS-Cre grading	Classification of PAS-Cre into cellular, cellular-fibrous and fibrous
MT-Cre grading	Classification of MT-Cre into cellular, cellular-fibrous and fibrous
PASM-Cre grading	Classification of PASM-Cre into cellular, cellular-fibrous and fibrous
PAS-MP grading	Classification of PAS-MP into segmental and global
PASM-MP grading	Classification of PASM-MP into segmental and global
PAS-EP grading	Classification of PAS-EP into segmental and global
PASM-EP grading	Classification of PASM-EP into segmental and global
Cross-modality Diagnosis	
Region classificatin	Distinguish capillary deposits from mesangial deposits
Pattern classification	Distinguish focal deposits from diffuse deposits
Few-shot learning for lesion recognition	
AIDPATH-Kidney-SVM	Distinguish normal and sclerotic glomeruli in AIDPATH-Kidney using SVM
AIDPATH-Kidney-LR	Distinguish normal and sclerotic glomeruli in AIDPATH-Kidney using LR
AIDPATH-Kidney-MLP	Distinguish normal and sclerotic glomeruli in AIDPATH-Kidney using MLP
AIDPATH-Kidney-RF	Distinguish normal and sclerotic glomeruli in AIDPATH-Kidney using RF
AIDPATH-Kidney-TPL	Distinguish normal and sclerotic glomeruli in AIDPATH-Kidney using TPL
XJ-IF-region-LR	Distinguish capillary deposits from mesangial deposits using LR
XJ-IF-region-MLP	Distinguish capillary deposits from mesangial deposits using MLP
XJ-IF-region-RF	Distinguish capillary deposits from mesangial deposits using RF
XJ-IF-region-TPL	Distinguish capillary deposits from mesangial deposits using TPL
XJ-IF-pattern-LR	Distinguish focal deposits from diffuse deposits using LR
XJ-IF-pattern-MLP	Distinguish focal deposits from diffuse deposits using MLP
XJ-IF-pattern-RF	Distinguish focal deposits from diffuse deposits using RF
XJ-IF-pattern-TPL	Distinguish focal deposits from diffuse deposits using TPL

Table S2: Number of glomeruli with each type of lesion in XJ-CLI.

Lesion	MH	PAS(+)	PAS(-)	Crum	ADH	SS	MN	MP	EP	Cre	GS	None of above lesions
n	1987	1441	24	119	511	90	611	5	1	132	954	9330

Table S3: Comparison of methods on lesion recognition in PAS staining (F1 score). The bold value indicates the best model.

Method	MH	PAS(+)	PAS(-)	Crum	ADH	SS	MN	MP	EP	Cre	GS
RandomInit	0.720	0.629	0.374	0.803	0.275	0.324	0.834	0.664	0.321	0.720	0.882
ImagenetPre	0.653	0.675	0.643	0.699	0.343	0.323	0.722	0.727	0.154	0.588	0.945
CONCH	0.782	0.870	0.990	0.683	0.686	0.942	0.896	0.923	0.950	0.932	0.955
PLIP	0.789	0.862	0.954	0.649	0.619	0.794	0.898	0.827	0.839	0.935	0.966
UNI	0.832	0.877	**0.999**	0.924	0.591	0.919	0.948	0.919	0.927	0.962	0.978
RenalPath	0.820	0.873	0.994	0.844	0.791	0.852	0.891	0.919	0.920	0.928	0.979
GloPath	**0.853**	**0.901**	0.998	**0.961**	**0.841**	**0.956**	**0.949**	**0.942**	**0.957**	**0.981**	**0.979**

Table S4: Comparison of methods on lesion recognition in MT staining (F1 score). The bold value indicates the best model.

Method	MH	ADH	SS	SFN	Cre	GS
RandomInit	0.735	0.441	0.515	0.768	0.636	0.790
ImagenetPre	0.663	0.623	0.393	0.725	0.508	0.791
CONCH	0.897	0.948	0.951	**0.978**	0.895	0.974
PLIP	0.891	**0.966**	0.907	0.853	0.885	0.955
UNI	0.883	0.895	**0.973**	0.974	0.955	0.975
RenalPath	0.917	0.928	0.895	0.974	0.911	0.977
GloPath	**0.935**	0.940	0.972	0.977	**0.980**	**0.984**

Table S5: Comparison of methods on lesion recognition in PASM staining (F1 score). The bold value indicates the best model.

Method	MH	PAS(-)	ADH	SS	SFN	MN	MP	EP	Cre	GS
RandomInit	0.530	0.548	0.624	0.695	0.738	0.791	0.805	0.890	0.798	0.890
ImagenetPre	0.610	0.381	0.530	0.863	0.753	0.408	0.698	0.848	0.907	0.838
CONCH	0.864	0.890	**0.954**	0.947	0.949	0.871	0.922	0.966	0.946	0.950
PLIP	0.815	0.981	0.890	0.896	0.814	0.893	0.923	0.946	0.963	0.950
UNI	0.877	0.986	0.894	0.964	0.966	0.855	0.920	**0.969**	0.958	0.959
RenalPath	0.887	0.982	0.774	0.919	0.960	0.862	0.901	0.957	0.946	0.966
GloPath	**0.895**	**0.990**	0.927	**0.965**	**0.970**	**0.927**	**0.928**	0.968	**0.970**	**0.971**

Table S6: Comparison of methods on AIDPATH-G under full supervision. The bold value indicates the best model.

Method	RandomInit	ImageNetPre	CONCH	PLIP	UNI	RenalPath	GloPath
ROC-AUC	0.878	0.893	0.992	0.990	0.999	0.995	**0.999**

Table S7: Comparison of methods on AIDPATH-G using SVM-based few-shot learning. The bold value indicates the best model.

Method	k=1	k=5	k=10	k=25	k=100
RandomInit	0.578	0.667	0.691	0.724	0.777
ImageNetPre	0.573	0.665	0.690	0.727	0.783
CONCH	0.606	0.771	0.828	0.876	0.960
PLIP	0.674	0.836	0.886	0.923	0.966
UNI	0.719	0.877	0.912	0.959	0.987
RenalPath	0.619	0.803	0.841	0.899	0.968
GloPath	**0.795**	**0.904**	**0.924**	**0.974**	**0.992**

Table S8: Comparison of methods on AIDPATH-G using LR-based few-shot learning. The bold value indicates the best model.

Method	k=1	k=5	k=10	k=25	k=100
RandomInit	0.578	0.677	0.781	0.835	0.886
ImageNetPre	0.573	0.682	0.783	0.839	0.890
CONCH	0.607	0.801	0.857	0.923	0.978
PLIP	0.674	0.853	0.910	0.946	0.979
UNI	0.716	0.896	0.934	0.969	0.989
RenalPath	0.619	0.823	0.884	0.933	0.976
GloPath	**0.796**	**0.921**	**0.944**	**0.976**	**0.992**

Table S9: Comparison of methods on AIDPATH-G using MLP-based few-shot learning. The bold value indicates the best model.

Method	k=1	k=5	k=10	k=25	k=100
RandomInit	0.577	0.662	0.762	0.821	0.887
ImageNetPre	0.573	0.675	0.768	0.831	0.893
CONCH	0.575	0.785	0.845	0.917	0.973
PLIP	0.662	0.861	0.908	0.941	0.976
UNI	0.717	0.873	0.915	0.964	0.984
RenalPath	0.641	0.813	0.891	0.933	0.975
GloPath	**0.771**	**0.904**	**0.925**	**0.974**	**0.989**

Table S10: Comparison of methods on AIDPATH-G using RF-based few-shot learning. The bold value indicates the best model.

Method	k=1	k=5	k=10	k=25	k=100
RandomInit	0.567	0.681	0.742	0.798	0.845
ImageNetPre	0.544	0.681	0.751	0.801	0.849
CONCH	0.515	0.762	0.828	0.875	0.931
PLIP	0.474	0.837	0.892	0.922	0.953
UNI	0.542	0.872	0.909	0.947	0.979
RenalPath	0.514	0.785	0.857	0.904	0.950
GloPath	**0.576**	**0.889**	**0.931**	**0.954**	**0.984**

Table S11: Comparison of methods on AIDPATH-G using PTL-based few-shot learning. The bold value indicates the best model.

Method	k=1	k=5	k=10	k=25	k=100
RandomInit	0.578	0.661	0.696	0.712	0.710
ImageNetPre	0.573	0.661	0.692	0.708	0.707
CONCH	0.606	0.783	0.827	0.838	0.856
PLIP	0.674	0.854	0.886	0.903	0.910
UNI	0.718	0.884	0.901	0.919	0.933
RenalPath	0.619	0.796	0.823	0.840	0.835
GloPath	**0.795**	**0.898**	**0.898**	**0.912**	**0.914**

Table S12: Comparison of methods on lesion grading (ROC-AUC). The bold value indicates the best model.

Method	PAS-MH-Grading	MT-MH-Grading	PASM-MH-Grading	PAS-Cre-Grading	MT-Cre-Grading	PASM-Cre-Grading	PAS-MP-Grading	PASM-MP-Grading	PAS-EP-Grading	PASM-EP-Grading
RandomInit	0.584	0.704	0.716	0.824	0.827	0.796	0.854	0.815	0.766	0.849
ImageNetPre	0.584	0.548	0.736	0.857	0.856	0.809	0.854	0.864	0.851	0.900
CONCH	0.700	0.866	0.849	0.927	0.954	0.931	0.910	0.849	0.932	**0.969**
PLIP	0.673	0.864	0.804	0.911	0.822	0.926	0.894	0.870	0.909	0.902
UNI	**0.736**	0.884	0.833	0.943	0.945	0.913	**0.920**	0.915	**0.954**	0.961
RenalPath	0.729	0.886	0.846	0.933	0.943	0.916	0.896	0.906	0.933	0.924
GloPath	0.726	**0.897**	**0.863**	**0.960**	**0.959**	**0.956**	0.902	**0.918**	0.950	0.962

Table S13: Comparison of methods on semantic segmentation of the glomerular bow and tuft. The bold value indicates the best model.

Method	Bow	Tuft
RandomInit	0.754	0.736
ImageNetPre	0.754	0.738
CONCH	0.754	0.768
PLIP	0.754	0.826
UNI	0.835	0.887
RenalPath	0.824	0.876
GloPath	**0.836**	**0.888**

Table S14: Comparison of significance level of pathomics-data mining of XJ-Light-1 for GloPath, UNI, and RenalPath.

Significance Level	***	**	*
GloPath	18	12	7
UNI	21	8	8
RenalPath	14	9	6

Table S15: Comparison of significance level of pathomics-data mining of KPMP-G for GloPath, UNI, and RenalPath.

Significance Level	***	**	*
GloPath	24	12	7
UNI	16	10	10
RenalPath	16	13	6

Table S16: Significance and effect size of clinicopathological correlation analysis for Gender on XJ-Light-1 (p-value (D))

Clinic Parameter: Gender			
Pathology Phenotype	GloPath	UNI	RenalPath
AreaBow_mean	0.0066 (0.1738)	0.0125 (0.1636)	0.0125 (0.1636)
AreaBow_med	0.0184 (0.1572)	0.0365 (0.1463)	0.0342 (0.1452)
AreaTuft_mean	0.0027 (0.1870)	0.0006 (0.1847)	0.0032 (0.2065)
AreaTuft_med	0.0456 (0.1410)	0.0371 (0.1661)	0.0107 (0.1449)
Ratio_mean	0.1220 (0.1210)	0.1731 (0.0812)	0.5433 (0.1131)
Ratio_med	0.3850 (0.0921)	0.2302 (0.1177)	0.1416 (0.1061)
CirBow_mean	0.4913 (0.0846)	0.4109 (0.0706)	0.7139 (0.0902)
CirBow_med	0.4620 (0.0866)	0.0884 (0.0570)	0.9043 (0.1279)
CirTuft_mean	0.5324 (0.0819)	0.0945 (0.1300)	0.0800 (0.1265)
CirTuft_med	0.5933 (0.0781)	0.0173 (0.1339)	0.0659 (0.1583)
EccBow_mean	0.9879 (0.0446)	0.9481 (0.0424)	0.9931 (0.0522)
EccBow_med	0.8543 (0.0611)	0.7352 (0.0545)	0.9294 (0.0692)
EccTuft_mean	0.7980 (0.0651)	0.8452 (0.0599)	0.8692 (0.0618)
EccTuft_med	0.9351 (0.0538)	0.3753 (0.0525)	0.9458 (0.0929)

Table S17: Significance and effect size of clinicopathological correlation analysis for Age on XJ-Light-1 (p-value (D))

Clinic Parameter: Age			
Pathology Phenotype	GloPath	UNI	RenalPath
AreaBow_mean	0.6849 (0.0791)	0.5131 (0.0661)	0.7777 (0.0827)
AreaBow_med	0.9145 (0.0556)	0.9608 (0.0669)	0.7636 (0.0500)
AreaTuft_mean	0.7726 (0.0664)	0.9135 (0.0669)	0.7631 (0.0556)
AreaTuft_med	0.2771 (0.1003)	0.5695 (0.0889)	0.4179 (0.0787)
Ratio_mean	0.0127 (0.1621)	0.1341 (0.1625)	0.0123 (0.1180)
Ratio_med	0.0996 (0.1245)	0.1303 (0.1122)	0.1725 (0.1187)
CirBow_mean	0.0393 (0.1427)	0.0375 (0.1112)	0.1795 (0.1437)
CirBow_med	0.0382 (0.1433)	0.0193 (0.0786)	0.5718 (0.1555)
CirTuft_mean	0.0086 (0.1682)	0.0691 (0.1450)	0.0347 (0.1319)
CirTuft_med	0.0030 (0.1840)	0.0244 (0.1976)	0.0012 (0.1512)
EccBow_mean	0.5043 (0.0831)	0.4434 (0.0776)	0.5909 (0.0874)
EccBow_med	0.0994 (0.1245)	0.4390 (0.0840)	0.4882 (0.0877)
EccTuft_mean	0.0304 (0.1473)	0.0639 (0.1584)	0.0160 (0.1335)
EccTuft_med	0.0420 (0.1414)	0.0122 (0.1783)	0.0044 (0.1627)

Table S18: Significance and effect size of clinicopathological correlation analysis for Creatinine on XJ-Light-1 (p-value (D))

Clinic Parameter: Creatinine			
Pathology Phenotype	GloPath	UNI	RenalPath
AreaBow_mean	0.1154 (0.1472)	0.3181 (0.1462)	0.1200 (0.1175)
AreaBow_med	0.0513 (0.1672)	0.0661 (0.1984)	0.0117 (0.1612)
AreaTuft_mean	0.1389 (0.1422)	0.1460 (0.1586)	0.0734 (0.1409)
AreaTuft_med	0.0548 (0.1656)	0.0524 (0.1969)	0.0127 (0.1667)
Ratio_mean	0.0419 (0.1718)	0.2449 (0.1469)	0.1169 (0.1259)
Ratio_med	0.1460 (0.1409)	0.1060 (0.1691)	0.0472 (0.1494)
CirBow_mean	0.9753 (0.0574)	0.0782 (0.0786)	0.7952 (0.1571)
CirBow_med	0.7473 (0.0823)	0.0218 (0.1296)	0.2164 (0.1859)
CirTuft_mean	0.0602 (0.1634)	0.2043 (0.1600)	0.0694 (0.1313)
CirTuft_med	0.1132 (0.1477)	0.0419 (0.1218)	0.2791 (0.1718)
EccBow_mean	0.9967 (0.0480)	0.9908 (0.0797)	0.7801 (0.0521)
EccBow_med	0.5091 (0.1004)	0.9252 (0.0994)	0.5221 (0.0659)
EccTuft_mean	0.4917 (0.1018)	0.6458 (0.0946)	0.5843 (0.0900)
EccTuft_med	0.8975 (0.0692)	0.4898 (0.0902)	0.6433 (0.1019)

Table S19: Significance and effect size of clinicopathological correlation analysis for IgA (Binary) on XJ-Light-1 (p-value (D))

Clinic Parameter: IgA (Binary)			
Pathology Phenotype	GloPath	UNI	RenalPath
AreaBow_mean	0.0007 (0.2240)	0.0002 (0.2156)	0.0012 (0.2438)
AreaBow_med	0.0091 (0.1838)	0.0098 (0.2059)	0.0023 (0.1824)
AreaTuft_mean	0.0041 (0.1969)	0.0027 (0.2062)	0.0023 (0.2033)
AreaTuft_med	0.0829 (0.1407)	0.0333 (0.1484)	0.0583 (0.1598)
Ratio_mean	0.5024 (0.0913)	0.3824 (0.0978)	0.4158 (0.1006)
Ratio_med	0.6412 (0.0817)	0.3629 (0.0777)	0.7009 (0.1022)
CirBow_mean	0.2376 (0.1146)	0.8695 (0.1125)	0.2565 (0.0652)
CirBow_med	0.3270 (0.1054)	0.7324 (0.0898)	0.5235 (0.0756)
CirTuft_mean	0.0158 (0.1739)	0.2263 (0.1511)	0.0513 (0.1160)
CirTuft_med	0.0243 (0.1659)	0.2270 (0.1677)	0.0221 (0.1159)
EccBow_mean	0.4487 (0.0953)	0.5257 (0.0913)	0.5031 (0.0897)
EccBow_med	0.5266 (0.0896)	0.8384 (0.1165)	0.2218 (0.0678)
EccTuft_mean	0.2027 (0.1189)	0.7522 (0.0949)	0.4534 (0.1742)
EccTuft_med	0.4899 (0.0922)	0.6155 (0.0617)	0.9073 (0.0834)

Table S20: Significance and effect size of clinicopathological correlation analysis for IgA Lee Score on XJ-Light-1 (p-value (ϵ^2))

Clinic Parameter: IgA Lee Score			
Pathology Phenotype	GloPath	UNI	RenalPath
AreaBow_mean	0.3151 (0.0008)	0.3200 (0.0033)	0.1986 (0.0008)
AreaBow_med	0.0028 (0.0266)	0.0034 (0.0320)	0.0010 (0.0254)
AreaTuft_mean	0.1219 (0.0060)	0.1274 (0.0083)	0.0805 (0.0058)
AreaTuft_med	0.0001 (0.0463)	0.0001 (0.0546)	0.0000 (0.0446)
Ratio_mean	0.0122 (0.0185)	0.0131 (0.0133)	0.0316 (0.0181)
Ratio_med	0.0009 (0.0327)	0.0010 (0.0278)	0.0022 (0.0321)
CirBow_mean	0.1823 (0.0038)	0.0022 (0.0000)	0.9849 (0.0279)
CirBow_med	0.0897 (0.0077)	0.0004 (0.0000)	0.8824 (0.0372)
CirTuft_mean	0.0054 (0.0230)	0.0033 (0.0052)	0.1421 (0.0257)
CirTuft_med	0.0014 (0.0303)	0.0040 (0.0058)	0.1271 (0.0243)
EccBow_mean	0.6610 (0.0000)	0.3627 (0.0000)	0.4726 (0.0000)
EccBow_med	0.6400 (0.0000)	0.3028 (0.0007)	0.3245 (0.0011)
EccTuft_mean	0.0413 (0.0119)	0.0727 (0.0064)	0.1140 (0.0088)
EccTuft_med	0.1139 (0.0064)	0.0617 (0.0074)	0.0950 (0.0097)

Table S21: Significance and effect size of clinicopathological correlation analysis for Disease on XJ-Light-1 (p-value (ϵ^2))

Clinic Parameter: Disease			
Pathology Phenotype	GloPath	UNI	RenalPath
AreaBow_mean	0.0000 (0.1013)	0.0000 (0.1051)	0.0000 (0.1038)
AreaBow_med	0.0000 (0.0652)	0.0000 (0.0688)	0.0000 (0.0172)
AreaTuft_mean	0.0000 (0.0972)	0.0000 (0.1099)	0.0000 (0.0980)
AreaTuft_med	0.0003 (0.0476)	0.0002 (0.0552)	0.0001 (0.0501)
Ratio_mean	0.0080 (0.0267)	0.0258 (0.0317)	0.0036 (0.0193)
Ratio_med	0.0010 (0.0395)	0.0011 (0.0528)	0.0001 (0.0388)
CirBow_mean	0.1014 (0.0102)	0.0208 (0.0000)	0.6053 (0.0207)
CirBow_med	0.0765 (0.0122)	0.0170 (0.0000)	0.9493 (0.0220)
CirTuft_mean	0.0144 (0.0230)	0.2787 (0.0054)	0.2017 (0.0030)
CirTuft_med	0.0240 (0.0198)	0.0112 (0.0098)	0.1080 (0.0246)
EccBow_mean	0.0697 (0.0128)	0.1483 (0.0107)	0.0947 (0.0076)
EccBow_med	0.0420 (0.0161)	0.2737 (0.0051)	0.2096 (0.0031)
EccTuft_mean	0.0413 (0.0162)	0.2053 (0.0086)	0.1275 (0.0052)
EccTuft_med	0.0687 (0.0129)	0.0340 (0.0089)	0.1229 (0.0175)

Table S22: Significance and effect size of clinicopathological correlation analysis for Lesion on XJ-Light-1 (p-value (ϵ^2))

	Clinic Parameter: Lesion		
Pathology Phenotype	GloPath	UNI	RenalPath
AreaBow_mean	0.0963 (0.0073)	0.0819 (0.0062)	0.1184 (0.0082)
AreaBow_med	0.8887 (0.0000)	0.8090 (0.0000)	0.9395 (0.0000)
AreaTuft_mean	0.4036 (0.0000)	0.3238 (0.0002)	0.3569 (0.0007)
AreaTuft_med	0.8736 (0.0000)	0.9567 (0.0000)	0.8917 (0.0000)
Ratio_mean	0.0273 (0.0141)	0.0359 (0.0152)	0.0226 (0.0126)
Ratio_med	0.0674 (0.0092)	0.1806 (0.0114)	0.0453 (0.0039)
CirBow_mean	0.0603 (0.0098)	0.0419 (0.0000)	0.7402 (0.0119)
CirBow_med	0.0795 (0.0083)	0.0813 (0.0000)	0.8173 (0.0082)
CirTuft_mean	0.0078 (0.0210)	0.0204 (0.0069)	0.1035 (0.0157)
CirTuft_med	0.0126 (0.0183)	0.0158 (0.0129)	0.0341 (0.0171)
EccBow_mean	0.8825 (0.0000)	0.8717 (0.0000)	0.8085 (0.0000)
EccBow_med	0.6739 (0.0000)	0.9555 (0.0000)	0.9018 (0.0000)
EccTuft_mean	0.2192 (0.0028)	0.3932 (0.0016)	0.2723 (0.0000)
EccTuft_med	0.4888 (0.0000)	0.3548 (0.0000)	0.4740 (0.0002)

Table S23: Multivariate Regression Analysis of Pathology Phenotypes Associated with Creatinine. Other dependent variables: Gender, Age, Disease Type (IgAN, LN, MN, DN, and MCD). G, A, D, and P indicate gender, age, disease type, and pathology phenotype respectively.

Pathology Phenotype	R^2	coef (G)	p (G)	coef (A)	p (A)	coef (D)	p (D)	coef (P)	p (P)
Proposed_AreaBow_mean	0.1071	23.1147	0.0135	1.5866	0.0000	-45.8914	0.0001	-0.0004	0.0476
Proposed_AreaBow_med	0.1188	23.4835	0.0113	1.5234	0.0000	-44.6479	0.0001	-0.0005	0.0031
Proposed_AreaTuft_mean	0.1202	25.1494	0.0070	1.5210	0.0000	-42.4377	0.0002	-0.0008	0.0023
Proposed_AreaTuft_med	0.1204	23.5983	0.0108	1.4747	0.0000	-44.5297	0.0001	-0.0006	0.0022
Proposed_Ratio_mean	0.1167	22.6231	0.0145	1.4406	0.0000	-46.7163	0.0000	-201.0923	0.0050
Proposed_Ratio_med	0.1262	22.6289	0.0139	1.4404	0.0000	-44.5344	0.0001	-219.8215	0.0006
Proposed_CirBow_mean	0.0978	21.0700	0.0242	1.6248	0.0000	-50.9139	0.0000	833.2359	0.6992
Proposed_CirBow_med	0.0980	21.0893	0.0240	1.6308	0.0000	-51.2372	0.0000	1027.1659	0.6443
Proposed_CirTuft_mean	0.1088	19.9190	0.0317	1.4999	0.0000	-51.0965	0.0000	-2921.3320	0.0320
Proposed_CirTuft_med	0.1140	19.4368	0.0356	1.4759	0.0000	-50.1649	0.0000	-3435.9837	0.0095
Proposed_EccBow_mean	0.0981	20.8299	0.0255	1.6174	0.0000	-50.7702	0.0000	-68.7994	0.6224
Proposed_EccBow_med	0.0980	20.9027	0.0250	1.6191	0.0000	-50.8867	0.0000	-56.3432	0.6425
Proposed_EccTuft_mean	0.0977	20.7669	0.0259	1.5903	0.0000	-50.7936	0.0000	39.3679	0.7597
Proposed_EccTuft_med	0.0994	20.6860	0.0264	1.5696	0.0000	-50.6826	0.0000	94.7958	0.3782

Table S24: Multivariate Regression Analysis of Pathology Phenotypes Associated with Disease Type. Other dependent variables: Gender, Age, Disease Type (IgAN, LN, MN, DN, and MCD).

Pathology Phenotype	R^2	coef (G)	p (G)	coef (A)	p (A)	coef (D)	p (D)	coef (P)	p (P)
Proposed_AreaBow_mean	0.1193	0.1964	0.5911	-0.0126	0.3174	-0.9831	0.1347	0.0000	0.0001
Proposed_AreaBow_med	0.1202	0.1415	0.6986	-0.0149	0.2406	-1.1577	0.0750	0.0000	0.0001
Proposed_AreaTuft_mean	0.1291	0.2466	0.5036	-0.0152	0.2349	-0.9330	0.1560	0.0000	0.0000
Proposed_AreaTuft_med	0.1125	0.1601	0.6600	-0.0158	0.2156	-1.2221	0.0587	0.0000	0.0001
Proposed_Ratio_mean	0.0660	0.1283	0.7201	-0.0134	0.2865	-1.3799	0.0299	-5.5755	0.0170
Proposed_Ratio_med	0.0763	0.1272	0.7241	-0.0133	0.2876	-1.3252	0.0375	-5.6022	0.0042
Proposed_CirBow_mean	0.0546	0.0252	0.9438	-0.0112	0.3667	-1.4670	0.0206	-117.2984	0.0994
Proposed_CirBow_med	0.0506	0.0383	0.9145	-0.0106	0.3898	-1.4466	0.0226	-95.1447	0.1872
Proposed_CirTuft_mean	0.0811	0.0453	0.8999	-0.0143	0.2596	-1.5632	0.0147	-136.2075	0.0019
Proposed_CirTuft_med	0.0905	0.0168	0.9632	-0.0150	0.2397	-1.5344	0.0181	-131.6665	0.0006
Proposed_EccBow_mean	0.0455	0.0724	0.8382	-0.0087	0.4733	-1.4987	0.0177	3.3006	0.5222
Proposed_EccBow_med	0.0514	0.0470	0.8950	-0.0098	0.4262	-1.4918	0.0184	6.0578	0.1703
Proposed_EccTuft_mean	0.0645	0.0591	0.8684	-0.0113	0.3613	-1.5036	0.0180	9.9814	0.0224
Proposed_EccTuft_med	0.0688	0.0552	0.8775	-0.0110	0.3735	-1.5086	0.0179	9.3923	0.0126

Table S25: Significance and effect size of clinicopathological correlation analysis for Enrollment Category on KPMP-G (p-value (ϵ^2))

Clinic Parameter: Enrollment Category			
Pathology Phenotype	GloPath	UNI	RenalPath
AreaBow_mean	0.0041 (0.0000)	0.0024 (0.0000)	0.0059 (0.0000)
AreaBow_med	0.6383 (0.0000)	0.5592 (0.0000)	0.6403 (0.0000)
AreaTuft_mean	0.1044 (0.0000)	0.2850 (0.0000)	0.0335 (0.0000)
AreaTuft_med	0.3037 (0.0000)	0.7116 (0.0000)	0.8076 (0.0000)
Ratio_mean	0.5496 (0.0000)	0.0072 (0.0000)	0.0457 (0.0000)
Ratio_med	0.3247 (0.0000)	0.0057 (0.0000)	0.2520 (0.0000)
CirBow_mean	0.0099 (0.0000)	0.2775 (0.0000)	0.3117 (0.0000)
CirBow_med	0.0120 (0.0000)	0.1452 (0.0000)	0.7453 (0.0000)
CirTuft_mean	0.0636 (0.0000)	0.0024 (0.0000)	0.0102 (0.0000)
CirTuft_med	0.0070 (0.0000)	0.0020 (0.0000)	0.1613 (0.0000)
EccBow_mean	0.6204 (0.0000)	0.8672 (0.0000)	0.5014 (0.0000)
EccBow_med	0.8631 (0.0000)	0.4035 (0.0000)	0.1044 (0.0000)
EccTuft_mean	0.6127 (0.0000)	0.7135 (0.0000)	0.7825 (0.0000)
EccTuft_med	0.3627 (0.0000)	0.7948 (0.0000)	0.3665 (0.0000)

Table S26: Significance and effect size of clinicopathological correlation analysis for Gender on KPMP-G (p-value (D))

Clinic Parameter: Gender			
Pathology Phenotype	GloPath	UNI	RenalPath
AreaBow_mean	0.5660 (0.0869)	0.9777 (0.0884)	0.5440 (0.0515)
AreaBow_med	0.9727 (0.0526)	0.7460 (0.0484)	0.9880 (0.0746)
AreaTuft_mean	0.9923 (0.0465)	0.9671 (0.0563)	0.9516 (0.0537)
AreaTuft_med	0.7014 (0.0777)	0.0800 (0.0909)	0.5088 (0.0142)
Ratio_mean	0.1904 (0.1207)	0.4768 (0.1238)	0.1689 (0.0932)
Ratio_med	0.3891 (0.1000)	0.2627 (0.1453)	0.0677 (0.0112)
CirBow_mean	0.0328 (0.1603)	0.1356 (0.1022)	0.3633 (0.1293)
CirBow_med	0.0111 (0.1805)	0.2411 (0.0935)	0.4736 (0.1143)
CirTuft_mean	0.3041 (0.1077)	0.0367 (0.0973)	0.4227 (0.1581)
CirTuft_med	0.1535 (0.1262)	0.0587 (0.1427)	0.0764 (0.1484)
EccBow_mean	0.7400 (0.0750)	0.5683 (0.0520)	0.9752 (0.0867)
EccBow_med	0.6525 (0.0810)	0.3592 (0.0727)	0.7735 (0.1026)
EccTuft_mean	0.5883 (0.0853)	0.9848 (0.0687)	0.8276 (0.0495)
EccTuft_med	0.9075 (0.0616)	0.7889 (0.0408)	0.9986 (0.0716)

Table S27: Significance and effect size of clinicopathological correlation analysis for Age on KPMP-G (p-value (ϵ^2))

Clinic Parameter: Age			
Pathology Phenotype	GloPath	UNI	RenalPath
AreaBow_mean	0.0591 (0.0185)	0.0607 (0.0165)	0.0747 (0.0182)
AreaBow_med	0.0125 (0.0313)	0.0189 (0.0268)	0.0217 (0.0280)
AreaTuft_mean	0.0256 (0.0255)	0.0320 (0.0274)	0.0204 (0.0236)
AreaTuft_med	0.0351 (0.0229)	0.1036 (0.0201)	0.0488 (0.0136)
Ratio_mean	0.3937 (0.0006)	0.0583 (0.0000)	0.4656 (0.0186)
Ratio_med	0.5305 (0.0000)	0.0892 (0.0000)	0.6226 (0.0149)
CirBow_mean	0.1248 (0.0119)	0.5318 (0.0000)	0.7286 (0.0000)
CirBow_med	0.4274 (0.0000)	0.3330 (0.0000)	0.8157 (0.0024)
CirTuft_mean	0.7107 (0.0000)	0.5707 (0.0000)	0.8937 (0.0000)
CirTuft_med	0.5385 (0.0000)	0.5693 (0.0000)	0.9221 (0.0000)
EccBow_mean	0.0634 (0.0179)	0.2042 (0.0161)	0.0774 (0.0073)
EccBow_med	0.0623 (0.0180)	0.3442 (0.0069)	0.2127 (0.0021)
EccTuft_mean	0.0623 (0.0180)	0.0316 (0.0359)	0.0070 (0.0237)
EccTuft_med	0.1209 (0.0122)	0.1140 (0.0211)	0.0433 (0.0127)

Table S28: Significance and effect size of clinicopathological correlation analysis for Proteinuria on KPMP-G (p-value (ϵ^2))

Clinic Parameter: Proteinuria			
Pathology Phenotype	GloPath	UNI	RenalPath
AreaBow_mean	0.0127 (0.0223)	0.0189 (0.0157)	0.0319 (0.0195)
AreaBow_med	0.2012 (0.0021)	0.1729 (0.0003)	0.2519 (0.0032)
AreaTuft_mean	0.0140 (0.0216)	0.0367 (0.0081)	0.0904 (0.0147)
AreaTuft_med	0.2247 (0.0012)	0.2766 (0.0000)	0.4925 (0.0000)
Ratio_mean	0.8597 (0.0000)	0.4209 (0.0000)	0.4607 (0.0000)
Ratio_med	0.7821 (0.0000)	0.7489 (0.0043)	0.1499 (0.0000)
CirBow_mean	0.4734 (0.0000)	0.6128 (0.0011)	0.2275 (0.0000)
CirBow_med	0.3488 (0.0000)	0.5162 (0.0000)	0.4318 (0.0000)
CirTuft_mean	0.7259 (0.0000)	0.3088 (0.0108)	0.0628 (0.0000)
CirTuft_med	0.7205 (0.0000)	0.2006 (0.0167)	0.0280 (0.0021)
EccBow_mean	0.8221 (0.0000)	0.8177 (0.0000)	0.6549 (0.0000)
EccBow_med	0.7867 (0.0000)	0.8618 (0.0000)	0.7874 (0.0000)
EccTuft_mean	0.3629 (0.0000)	0.4663 (0.0000)	0.5441 (0.0000)
EccTuft_med	0.5128 (0.0000)	0.3355 (0.0000)	0.7193 (0.0000)

Table S29: Significance and effect size of clinicopathological correlation analysis for A1c on KPMP-G (p-value (ϵ^2))

Clinic Parameter: A1c			
Pathology Phenotype	GloPath	UNI	RenalPath
AreaBow_mean	0.0001 (0.0574)	0.0003 (0.0506)	0.0003 (0.0523)
AreaBow_med	0.0011 (0.0429)	0.0008 (0.0405)	0.0015 (0.0450)
AreaTuft_mean	0.0007 (0.0453)	0.0007 (0.0440)	0.0009 (0.0458)
AreaTuft_med	0.0051 (0.0320)	0.0132 (0.0344)	0.0035 (0.0252)
Ratio_mean	0.5624 (0.0000)	0.0639 (0.0000)	0.6657 (0.0139)
Ratio_med	0.3730 (0.0004)	0.0300 (0.0000)	0.6526 (0.0194)
CirBow_mean	0.9727 (0.0000)	0.4376 (0.0000)	0.7993 (0.0000)
CirBow_med	0.7370 (0.0000)	0.7429 (0.0000)	0.7916 (0.0000)
CirTuft_mean	0.0923 (0.0112)	0.1097 (0.0000)	0.7729 (0.0099)
CirTuft_med	0.1790 (0.0062)	0.0804 (0.0000)	0.7616 (0.0122)
EccBow_mean	0.0406 (0.0172)	0.0485 (0.0230)	0.0181 (0.0159)
EccBow_med	0.0548 (0.0150)	0.3759 (0.0274)	0.0097 (0.0003)
EccTuft_mean	0.0995 (0.0106)	0.0289 (0.0078)	0.1456 (0.0196)
EccTuft_med	0.1737 (0.0064)	0.0197 (0.0173)	0.0398 (0.0224)

Table S30: Significance and effect size of clinicopathological correlation analysis for Albuminuria on KPMP-G (p-value (D))

Clinic Parameter: Albuminuria			
Pathology Phenotype	GloPath	UNI	RenalPath
AreaBow_mean	0.0002 (0.2975)	0.0004 (0.2859)	0.0005 (0.2885)
AreaBow_med	0.0467 (0.1907)	0.0263 (0.1842)	0.0596 (0.2049)
AreaTuft_mean	0.0003 (0.2949)	0.0010 (0.2476)	0.0037 (0.2717)
AreaTuft_med	0.1949 (0.1494)	0.1971 (0.1343)	0.3000 (0.1490)
Ratio_mean	0.8728 (0.0804)	0.8504 (0.1389)	0.2643 (0.0828)
Ratio_med	0.7901 (0.0886)	0.3085 (0.1528)	0.1756 (0.1333)
CirBow_mean	0.0329 (0.1995)	0.7690 (0.1977)	0.0354 (0.0906)
CirBow_med	0.0745 (0.1782)	0.4520 (0.1684)	0.1051 (0.1180)
CirTuft_mean	0.0242 (0.2069)	0.0824 (0.2516)	0.0030 (0.1754)
CirTuft_med	0.1637 (0.1550)	0.0696 (0.2704)	0.0011 (0.1800)
EccBow_mean	0.4869 (0.1149)	0.3201 (0.1560)	0.1586 (0.1319)
EccBow_med	0.1777 (0.1524)	0.7709 (0.1474)	0.2068 (0.0904)
EccTuft_mean	0.7293 (0.0940)	0.6466 (0.1105)	0.5366 (0.1010)
EccTuft_med	0.8199 (0.0858)	0.8728 (0.1157)	0.4784 (0.0804)

Table S31: Significance and effect size of clinicopathological correlation analysis for Diabetes History on KPMP-G (p-value (D))

Clinic Parameter: Diabetes History			
Pathology Phenotype	GloPath	UNI	RenalPath
AreaBow_mean	0.0004 (0.2446)	0.0006 (0.2297)	0.0012 (0.2394)
AreaBow_med	0.0085 (0.1964)	0.0158 (0.1466)	0.0939 (0.1851)
AreaTuft_mean	0.0239 (0.1767)	0.1065 (0.1485)	0.0869 (0.1435)
AreaTuft_med	0.1842 (0.1292)	0.3436 (0.1231)	0.2290 (0.1103)
Ratio_mean	0.5747 (0.0914)	0.9438 (0.1464)	0.0946 (0.0609)
Ratio_med	0.4944 (0.0976)	0.8057 (0.0794)	0.7468 (0.0748)
CirBow_mean	0.0481 (0.1620)	0.7128 (0.0755)	0.7959 (0.0815)
CirBow_med	0.0204 (0.1801)	0.9922 (0.1145)	0.3033 (0.0495)
CirTuft_mean	0.4785 (0.0991)	0.4438 (0.1106)	0.3399 (0.1014)
CirTuft_med	0.6729 (0.0847)	0.0681 (0.0796)	0.7440 (0.1542)
EccBow_mean	0.0030 (0.2146)	0.0076 (0.2212)	0.0020 (0.1988)
EccBow_med	0.0121 (0.1902)	0.0553 (0.2097)	0.0041 (0.1590)
EccTuft_mean	0.0083 (0.1967)	0.0066 (0.1586)	0.0563 (0.2008)
EccTuft_med	0.0110 (0.1917)	0.1413 (0.1621)	0.0479 (0.1362)

Table S32: Significance and effect size of clinicopathological correlation analysis for Hypertension History on KPMP-G (p-value (D))

Clinic Parameter: Hypertension History			
Pathology Phenotype	GloPath	UNI	RenalPath
AreaBow_mean	0.3550 (0.1368)	0.4629 (0.1098)	0.6268 (0.1252)
AreaBow_med	0.3560 (0.1367)	0.4251 (0.1368)	0.3550 (0.1290)
AreaTuft_mean	0.9289 (0.0768)	0.9085 (0.1069)	0.6563 (0.0817)
AreaTuft_med	0.1961 (0.1596)	0.1222 (0.1559)	0.2163 (0.1752)
Ratio_mean	0.0075 (0.2485)	0.0041 (0.3143)	0.0003 (0.2609)
Ratio_med	0.0268 (0.2179)	0.0007 (0.2186)	0.0256 (0.2954)
CirBow_mean	0.0173 (0.2292)	0.2707 (0.1095)	0.6311 (0.1478)
CirBow_med	0.0023 (0.2724)	0.4071 (0.1867)	0.0848 (0.1306)
CirTuft_mean	0.0143 (0.2334)	0.0322 (0.2876)	0.0011 (0.2137)
CirTuft_med	0.0138 (0.2338)	0.0167 (0.2261)	0.0190 (0.2296)
EccBow_mean	0.6984 (0.1031)	0.8321 (0.1140)	0.5798 (0.0906)
EccBow_med	0.9888 (0.0637)	0.6152 (0.1143)	0.5755 (0.1106)
EccTuft_mean	0.1422 (0.1708)	0.1106 (0.1712)	0.1389 (0.1787)
EccTuft_med	0.7903 (0.0947)	0.1666 (0.1877)	0.0795 (0.1645)

Table S33: Significance and effect size of clinicopathological correlation analysis for eGFR on KPMP-G (p-value (ϵ^2))

Clinic Parameter: eGFR			
Pathology Phenotype	GloPath	UNI	RenalPath
AreaBow_mean	0.9834 (0.0000)	0.9294 (0.0000)	0.8818 (0.0000)
AreaBow_med	0.0972 (0.0086)	0.1308 (0.0100)	0.0784 (0.0067)
AreaTuft_mean	0.2920 (0.0015)	0.0550 (0.0042)	0.1933 (0.0123)
AreaTuft_med	0.0240 (0.0177)	0.0041 (0.0239)	0.0093 (0.0291)
Ratio_mean	0.0006 (0.0412)	0.0000 (0.0642)	0.0000 (0.0844)
Ratio_med	0.0002 (0.0481)	0.0000 (0.0636)	0.0000 (0.0784)
CirBow_mean	0.0025 (0.0324)	0.2314 (0.0027)	0.2435 (0.0030)
CirBow_med	0.0046 (0.0285)	0.1615 (0.0053)	0.1629 (0.0053)
CirTuft_mean	0.0100 (0.0234)	0.0003 (0.0173)	0.0258 (0.0463)
CirTuft_med	0.0181 (0.0195)	0.0004 (0.0115)	0.0628 (0.0440)
EccBow_mean	0.6074 (0.0000)	0.6184 (0.0005)	0.3383 (0.0000)
EccBow_med	0.3967 (0.0000)	0.2768 (0.0000)	0.3666 (0.0018)
EccTuft_mean	0.1259 (0.0070)	0.0494 (0.0093)	0.0879 (0.0130)
EccTuft_med	0.1903 (0.0043)	0.0588 (0.0050)	0.1711 (0.0119)

Table S34: Comparison of methods on XJ-IF for cross-modality diagnosis based on full supervision.

Method	RandomInit	ImageNetPre	CONCH	PLIP	UNI	RenalPath	GloPath
Region	0.548	0.508	0.850	0.836	0.917	0.914	**0.942**
Pattern	0.473	0.479	0.801	0.849	0.928	0.912	**0.944**

Table S35: Comparison of methods on deposition region classification on XJ-IF using LR-based few-shot learning. The bold value indicates the best model and the hyphen mean the metrics value is lower than 0.5 and not applicable.

Method	k=1	k=5	k=10	k=25	k=100
RandomInit	-	-	-	0.501	-
ImageNetPre	-	-	-	-	-
CONCH	-	-	-	-	0.823
PLIP	-	-	-	0.561	0.848
UNI	-	-	0.704	0.859	0.929
RenalPath	-	-	0.657	0.685	0.862
GloPath	**-**	**-**	**0.784**	**0.911**	**0.945**

Table S36: Comparison of methods on deposition pattern classification on XJ-IF using LR-based few-shot learning. The bold value indicates the best model and the hyphen mean the metrics value is lower than 0.5 and not applicable.

Method	k=1	k=5	k=10	k=25	k=100
RandomInit	-	**0.500**	0.503	-	0.505
ImageNetPre	-	-	0.503	0.500	0.505
CONCH	-	-	-	-	0.804
PLIP	-	-	-	0.520	0.825
UNI	-	-	0.679	0.854	0.922
RenalPath	-	-	0.674	0.694	0.859
GloPath	**-**	**-**	**0.852**	**0.904**	**0.941**

Table S37: Comparison of methods on deposition region classification on XJ-IF using MLP-based few-shot learning. The bold value indicates the best model and the hyphen mean the metrics value is lower than 0.5 and not applicable.

Method	k=1	k=5	k=10	k=25	k=100
RandomInit	0.511	0.514	0.521	0.525	0.550
ImageNetPre	0.512	0.512	0.521	0.519	0.544
CONCH	0.560	0.635	0.719	0.794	0.868
PLIP	0.610	0.733	0.797	0.840	0.880
UNI	0.625	0.717	0.830	0.885	**0.939**
RenalPath	0.660	0.739	0.792	0.852	0.901
GloPath	**0.666**	**0.764**	**0.851**	**0.903**	0.937

Table S38: Comparison of methods on deposition pattern classification on XJ-IF using MLP-based few-shot learning. The bold value indicates the best model and the hyphen mean the metrics value is lower than 0.5 and not applicable.

Method	k=1	k=5	k=10	k=25	k=100
RandomInit	0.500	0.507	0.510	0.516	0.538
ImageNetPre	0.500	0.507	0.507	0.512	0.532
CONCH	0.536	0.663	0.695	0.753	0.852
PLIP	0.663	0.758	0.762	0.779	0.864
UNI	0.618	0.787	0.822	0.868	0.924
RenalPath	0.662	0.761	0.790	0.817	0.893
GloPath	**0.665**	**0.835**	**0.843**	**0.887**	**0.926**

Table S39: Comparison of methods on deposition region classification on XJ-IF using RF-based few-shot learning. The bold value indicates the best model and the hyphen mean the metrics value is lower than 0.5 and not applicable.

Method	k=1	k=5	k=10	k=25	k=100
RandomInit	0.511	0.516	0.525	0.532	0.553
ImageNetPre	0.512	0.515	0.518	0.513	0.507
CONCH	0.556	0.624	0.705	0.788	0.869
PLIP	0.608	0.730	0.796	0.830	0.876
UNI	0.626	0.706	0.815	0.879	0.939
RenalPath	0.664	0.749	0.786	0.845	0.898
GloPath	**0.668**	**0.757**	**0.843**	**0.905**	**0.945**

Table S40: Comparison of methods on deposition pattern classification on XJ-IF using RF-based few-shot learning. The bold value indicates the best model and the hyphen mean the metrics value is lower than 0.5 and not applicable.

Method	k=1	k=5	k=10	k=25	k=100
RandomInit	-	0.509	0.513	0.512	0.514
ImageNetPre	-	0.501	0.508	0.507	0.510
CONCH	0.537	0.659	0.687	0.741	0.852
PLIP	0.658	0.759	0.752	0.774	0.860
UNI	0.612	0.783	0.813	0.864	0.927
RenalPath	0.667	0.769	0.785	0.812	0.889
GloPath	**0.645**	**0.834**	**0.843**	**0.896**	**0.939**

Table S41: Comparison of methods on deposition region classification on XJ-IF using PTL-based few-shot learning. The bold value indicates the best model and the hyphen mean the metrics value is lower than 0.5 and not applicable.

Method	k=1	k=5	k=10	k=25	k=100
RandomInit	0.506	0.514	0.529	0.541	0.556
ImageNetPre	0.499	0.515	0.531	0.536	0.558
CONCH	0.552	0.610	0.687	0.751	0.841
PLIP	0.592	0.722	0.797	0.827	0.860
UNI	0.593	0.682	0.791	0.850	0.908
RenalPath	0.592	0.721	0.791	0.833	0.863
GloPath	**0.614**	**0.725**	**0.820**	**0.886**	**0.928**

Table S42: Comparison of methods on deposition pattern classification on XJ-IF using PTL-based few-shot learning. The bold value indicates the best model and the hyphen mean the metrics value is lower than 0.5 and not applicable.

Method	k=1	k=5	k=10	k=25	k=100
RandomInit	0.508	0.508	0.517	0.526	0.559
ImageNetPre	0.503	0.508	0.512	0.520	0.555
CONCH	0.537	0.640	0.660	0.708	0.818
PLIP	**0.653**	0.757	0.764	0.782	0.842
UNI	0.634	0.759	0.794	0.841	0.907
RenalPath	0.620	0.754	0.771	0.805	0.862
GloPath	0.558	**0.781**	**0.793**	**0.876**	**0.929**

Table S43: Performance of GloPath in large-scale real-world study.

Lesion	MH	PAS(+)	PAS(-)	Crum	ADH	SS	MN	MP	EP	Cre	GS
ROC-AUC	0.897	0.857	0.983	0.747	0.790	0.920	0.919	0.982	0.999	0.954	0.997

Table S44: Details of the glomerular lesion annotation on XJ-Light-1.

Staining	MH	PAS(+)	PAS(-)	Crum	ADH	SS	SFN	MN	MP	EP	Cre	GS
PAS	905	479	75	31	222	76	-	101	189	69	110	263
MT	282	-	-	-	39	30	67	-	-	-	38	128
PASM	298	-	73	-	44	34	38	216	132	64	114	164

Table S45: Details of the IF markers in XJ-IF.

Marker	IgG	IgG2	IgA	IgG4	PLA2R	IgG1	Lambda	C4	C3	C1q	IgG3	Kappa	IgM	AA	Fibrin	IgG-PLA2R1	Albumin	THSD7A	HBsAg	HBeAg	IgG14	[illegible]
Unlabeled	328	4	304	234	136	159	71	9	57	43	4	36	11		3		17	12	23	18	3	[illegible]
Labeled	366	31	353	337	178	149	51	6	81	58	22	26	39	10	3	1						

Table S46: Details of the annotated images on XJ-IF.

Class	Region		Pattern	
	Capillary	Membrane	Scatted	Diffused
Number	1053	658	747	962

Table S47: Details of the clinical variables on XJ-Light-1.

Clinical Variable	Groups
Gender	Male, Female
Age(years old)	>43, ≤43
Creatinine(umol/L)	>59, ≤59
IgA(Binary)	non-IgA, IgA
IgA Lee Score	II, III, IV
Disease	IgAN, LN, MN, DN, MCD
Lesion	0-Mild, 1-Moderatre, 2-Severe

Table S48: Details of the clinical variables on KPMP-G.

Clinical Variable	Groups
Enrollment Category	CKD, AKI, DM-R
Gender	Male, Female
Age(years old)	<30, 30-39, 40-49, 50-59, 60-69, ≥70
Proteinuria(mg)	<150, 150-500, 500-1000, ≥1000
A1c(%)	<6.5, 6.5-7.5, 7.5-8.5, ≥8.5
Albuminuria(mg/L)	<300, ≥300
Diabetes History	Yes, No
Hypertension History	Yes, No
eGFR(mL/min/1.73mˆ2)	<50, 50-100, >100

Table S49: Details of the morphological variables. S, P, a, and b refer to the area enclosed by the contour, the perimeter, the major axis, and the minor axis of the ellipse fitted to the contour of the bow or tuft segmented by the model, respectively.

Morphological Variable	Formulation (Glomerular-level)	Definition (case-level)
AreaBow_mean	S_{bow}	Mean of all the S_{bow}
AreaBow_med		Median of all the S_{bow}
AreaTuft_mean	S_{tuft}	Mean of all the S_{tuft}
AreaTuft_med		Median of all the S_{tuft}
Ratio_mean	$R = \frac{S_{tuft}}{S_{bow}}$	Mean of all the R
Ratio_med		Median of all the R
CirBow_mean	$Cir_{bow} = \frac{4\pi S_{bow}}{P_{bow}^2}$	Mean of all the Cir_{bow}
CirBow_med		Median of all the Cir_{bow}
CirTuft_mean	$Cir_{tuft} = \frac{4\pi S_{tuft}}{P_{tuft}^2}$	Mean of all the Cir_{tuft}
CirTuft_med		Median of all the Cir_{tuft}
EccBow_mean	$Ecc_{bow} = (1 - \frac{b_{bow}^2}{a_{bow}^2})^{\frac{1}{2}}$	Mean of all the Ecc_{bow}
EccBow_med		Median of all the Ecc_{bow}
EccTuft_mean	$Ecc_{tuft} = (1 - \frac{b_{tuft}^2}{a_{tuft}^2})^{\frac{1}{2}}$	Mean of all the Ecc_{tuft}
EccTuft_med		Median of all the Ecc_{tuft}
